# Supplementary material for: Concerted transcriptional regulation of the morphogenesis of hypothalamic neurons by ONECUT3
Source: Nat Commun. 2024 Oct 5;15:8631. doi: 10.1038/s41467-024-52762-z (PMC11452682; doi:10.1038/s41467-024-52762-z)

Supplementary Information File to the Manuscript NCOMMS-22-47020D:

Concerted transcriptional regulation of the morphogenesis of hypothalamic neurons by ONECUT3

Maja Zupančič, Erik Keimpema, Evgenii O. Tretiakov, Stephanie J. Eder, Itamar Lev, Lukas Englmaier, Pradeep Bhandari, Simone A. Fietz, Wolfgang Härtig, Estelle Renaux, Andreas Villunger, Tomas Hökfelt, Manuel Zimmer, Frédéric Clotman and Tibor Harkany

Contents:

- Supplementary tables 1 – 8,
- Supplementary figures 1 – 11,
- Legends to Supplementary tables and figures,
- Supplementary references.

Tables

Supplementary Table 1: Description of mouse strains

Abbreviated name	Strain name	Source
GAD67-GFP	GAD67$^{GFP/+}$	Yuchio Yanagawa[1]
GAD65-GFP	GAD65$^{GFP/+}$	Gábor Szabó[2]
Onecut3-iCre(3)	(BAC)Onecut3$^{iCre/+}$	Ferenc Erdélyi, Gábor Szabó[3]
Onecut3-mCherry(28)	(BAC)-Onecut3$^{mCherry/+}$	Ferenc Erdélyi, Gábor Szabó[3]
TRH-tdTomato	TRH-Ires-tdTomato	Csaba Fekete[4]
Tau-mGFP	Tau$^{mGFP/+}$	Jackson Laboratory, strain #021162[5]
Ai14	Ai14-tdTomato	Jackson Laboratory, strain #007909[6]

Supplementary Table 2: Primer pairs for genotyping of mice

Transgenic animal	Genotyping primer pairs
GAD67-GFP, GAD65-GFP, Tau-mGFP	Forward: 5'-AAGTTCATCTGCACCACC-3'<br>Reverse: 5'-TCCTTGAAGAAGATGGTGCG-3'
Onecut3-iCre(3)	Forward: 5'-AGATGCCAGGACATCAGGAACCTG-3'<br>Reverse: 5'-ATCAGCCACACCAGACACAGAGATC-3'
Onecut3-mCherry(28)	Forward: 5'-AGGACGGCGAGTTCATCTAC-3'<br>Reverse: 5'-TGGTGTAGTCCTCGTTGTGG-3'
Ai14, TRH-TdTomato	Forward: 5'-CTGTTCCTG TACGGCATGG-3'<br>Reverse: 5'-GGCATTAAAGCAGCGTATCC-3'

Supplementary Table 3: *C. elegans* strain description

Strain name	Description	Source
N2	wild-type	CGC, USA
ceh-48 (tm237)	AT insertion and a 501 bp deletion in *ceh-48* gene	NBP, Japan
unc-53 (MT152)	320 bp deletion in *unc-53* gene	CGC, USA

Supplementary Table 4: qPCR primers for mice

Target gene	Primer pairs
Onecut3	Forward: 5'-GCTGATTGCCATCTTCAAGG-3'<br>Reverse: 5'-GAAGTTGCTGACAGTGTTGA-3'
Nav2	Forward: 5'-AAGCTCAGGTGCTTGGTGTT-3'<br>Reverse: 5'-AGCCCAAGACGTATCAACCG-3'
Tbp	Forward: 5'-CCTTGTACCCTTCACCAATGAC-3'<br>Reverse: 5'-ACAGCCAAGATTCACGGTAGA-3'

Supplementary Table 5: qPCR primer pairs for *C. elegans*

Target gene	Primer pairs
pmp-3	Forward: 5’-ATTGCACATCCCGCATGGA-3’<br>Reverse: 5’-GAGGCGTTTTTGCGACCTTT-3’
unc-53	Forward: 5’-GAGTTCACATCCACCGAGCA-3’<br>Reverse: 5’-GCCGAGTAGGATCCTGAAGAA-3’

Supplementary Table 6: Immunoreagents, their use, and sources

Antibody	Host species	Concentration	Source
Acetylated tubulin	mouse	1:1,000 (ICC)	Sigma, #T7451
CPCA-mCherry	chicken	1:1,000 (IHC, ICC)	EnCor, #CPCA-mCherry
Doublecortin	guinea pig	1:1,000 (ICC)	Millipore, #AB2253
GAP43	rabbit	1:500 (IHC)	Millipore, #AB5220
GFAP	rabbit	1:1,000 (IHC)	Synaptic Sys., #173002
GFP-FITC	goat	1:1,000 (IHC)	Abcam, #ab6662
Hoechst 33342	-	1:10,000 (IHC, ICC)	Sigma, #14533
MAP2	guinea pig	1:1,000 (ICC)	Synaptic Sys. #188004
NAV2	rabbit	1:1,000 (IHC), 1:250 (ICC)	Novus, #NBP1-84615
NeuN	mouse	1:1,000 (IHC)	Millipore, #MAB377
Onecut1	sheep	1:50 (IHC)	R&D, #AF6277
Onecut2	sheep	1:50 (IHC)	R&D, #AF6294
Onecut3	guinea pig	1:5,000 (IHC), 1:2,000 (ICC)	F. Clotman[7]
Phalloidin-647	*A. phalloides*	1:500 (ICC)	Thermo Fisher, #A30107
Phalloidin-555	*A. phalloides*	1:500 (ICC)	Thermo Fisher, #A34055
P-histone H3	rabbit	1:500 (ICC)	Cell Signalling, #9701
Pro-TRH	rat	1:200 (IHC)	C. Fekete[8]
Sox2	rabbit	1:500 (IHC)	Abcam, #ab97959
TH	rabbit	1:500 (IHC, ICC)	Millipore, #AB152
TUJ1	mouse	1:2,000 (ICC)	Promega, # G7121

Supplementary Table 7: Primer pairs for select genes in Neuro-2a cells

Target gene	Primer pairs
Onecut3	Forward:5'-GCTGATTGCCATCTTCAAGG-3'
	Reverse: 5'-GAAGTTGCTGACAGTGTTGA-3'
Gad1	Forward: 5'-TGGTGATGGGATATT-3'
	Reverse: 5'-ACTTGTAACCAGCAG-3'
Gad2	Forward: 5'-TTCAGTACGTGGTGA-3'
	Reverse: 5'-TGCCAATTCCCAATT-3'
Th	Forward: 5'-TGTTTCAGTGCACACAGTAC-3'
	Reverse: 5'-CCAATGTCCTGGGAGAACTG-3'
Trh	Forward: 5'-TGGTGCTGCCTTAGATTCCT-3'
	Reverse: 5'-GTCGTTTGTGGGGTCTCCAG-3'

Supplementary Table 8: Statistical differences for amphid neurons in *C. elegans* mutants

Position	wild-type *vs ceh-48*	wild-type *vs. unc-53*
Anterior	$9.63E^{-05}$	$1.32182E^{-05}$
Lateral	0.001484	$8.82251E^{-05}$
Total neurons	0.495147	0.596547075

Legends to Supplementary Data Figures

Supplementary Figure 1. *In utero* electroporation at E13.5. (a, b) *In utero* electroporation was performed with an mCherry plasmid at embryonic day (E)13.5. Tissues were collected at either E15.5 (a) or E18.5 (b). Immunohistochemistry showed a lack of co-labelling of mCherry signal (open arrowheads) and ONECUT3 protein (green), suggesting that ONECUT3 is only expressed in postmitotic progeny and not in progenitor cells lining the wall of the 3rd ventricle (3V). Scale bars = 100 µm (a, b).

Supplementary Figure 2. ONECUT transcription factors in the developing hypothalamus. (a,b) *Onecut1-3* expression in the mouse hypothalamus at embryonic day (E)14.5, as shown by *in situ* hybridization (a) and immunohistochemistry (b,b_1). (c) *In situ* hybridization at postnatal day (P)3 revealed that the pattern of *Onecut1-3* mRNA distribution is unchanged compared to fetal development. (d,d_1) ONECUT1/2 (green) and ONECUT3 (red) proteins were also detected on P3. (e-f_1) The localization of *Onecut1-3* mRNAs were unchanged in adulthood (P32) relative to earlier developmental stages. However, ONECUT1/2 protein were no longer detected. Abbreviations: 3V, third ventricle; AHA, anterior hypothalamic area; LH, lateral hypothalamus; OC1, *Onecut1*; OC2, *Onecut2*; OC3, *Onecut3*; PeVN, periventricular nucleus. Asterisks denote prospective hypothalamic structures. Scale bars = 100 µm (a-f), 20 µm (insets from a-e).

Supplementary Figure 3. Phylogenetic conservation of ONECUT3 expression in hypothalamus. (a) Phylogenetic tree of some evolutionary segregating mammals. Animals used in this study were marked in red. (b-b_2) Expression pattern of ONECUT3 (OC3) protein in adult mouse anterior (b) and tuberal (b_1) hypothalamus. OC3 was expressed exclusively in neurons (NeuN$^+$, arrowheads). (c-i) Immunohistochemistry for OC3 across different species. OC3 expression was conserved in naked mole rat (c,c_1), Seba's fruit bat (d,f), Indian flying fox (e), wild boar (g), sheep (h) and mouse lemur (i). The age range of the subjects was given throughout (E, embryonic day; M, months; y, year). (j-j_1) OC3 expression in human fetal hypothalamus (20 weeks + 6 days of gestation). Arrowheads mark ONECUT3$^+$ neurons. Abbreviations: 3V, third ventricle; AHA, anterior hypothalamic area; CPu, caudate putamen; CTX, cortex; DMH, dorsomedial nucleus; HYP, hypothalamus; LV, lateral ventricle; PeVN, periventricular nucleus; SCN, suprachiasmatic nucleus; TH, tyrosine hydroxylase; VMH, ventromedial nucleus. Scale bars = 1 mm (j), 100 µm (b,b1,c-i,j_1).

Supplementary Figure 4. Spatiotemporal mapping of ONECUT3 mRNA expression in the hypothalamus. (a,a_1) ONECUT3 expression demarcated cell contingents that form the prospective periventricular (PeVN), anterior (AHA) and lateral hypothalamic (LH) nuclei in sections spanning the anterior (a) and tuberal (a_1) hypothalamus at E14.5. ONECUT3$^+$/TH$^+$ neurons were seen along the midline, in the prospective PeVN. (a_2) Schema of the ONECUT3$^+$ cell continuum in the fetal hypothalamus, including dopamine cell identity. Upper and lower panels correspond to the anterior and tuberal regions, respectively. (b,b_1) ONECUT3 distribution on postnatal day (P)3. Sections that corresponded to those

shown in (a,a_1) were selected. Note the recruitment of ONECUT3$^+$ cells to their final locations within the nuclei described for E14.5. (b_2) A medial-to-lateral cell continuum enriched in neurons was found, with ONECUT3$^+$/TH$^+$ neurons (solid arrowheads) limited to the midline. Open arrowheads label ONECUT3$^+$/TH- neurons. (b_2) Shema of cell distribution in the neonatal hypothalamus, corresponding to a_2. Abbreviations: 3V, third ventricle; ARC, arcuate nucleus; DMH, dorsomedial hypothalamic nucleus; SCN, suprachiasmatic nucleus; VMH, ventromedial hypothalamus; TU, tuberal nucleus. Regions labeled with asterisks are putative anatomical areas early in development. Scale bars = 100 µm (a-b_2), 20 µm (insets in a-b_1).

Supplementary Figure 5. *Onecut3* mRNA expression within GABA and glutamate-dominated hypothalamic territories. *In situ* hybridization was performed for *Onecut3* (red), vesicular GABA transporter (*Slc32a1*; blue) and vesicular glutamate transporter 2 (*Slc17a6*; green). (a) ON postnatal day (P)3, both *Onecut3$^+$/Slc32a1$^+$* (blue arrowheads) and *Onecut3$^+$/Slc17a6$^+$* (green arrowheads) were found, with *Onecut3$^+$/Slc32a1$^+$* cells distributed along the midline while *Onecut3$^+$/Slc17a6$^+$* neurons positioned laterally (a1-4’). (b) The dichotomy of neurochemical identity was maintained in adulthood. Abbreviations: 3V, third ventricle; AHA, anterior hypothalamic area; DMH, dorsomedial hypothalamus; LPO, lateral preoptic area; LH, lateral hypothalamus; MPO, medial preoptic area; PeVN, periventricular nucleus; RCH, retrochiasmatic nucleus; VMH, ventromedial hypothalamus; TU, tuberal nucleus. Scale bars = 100 µm (a-b_2), 20 µm (all insets).

Supplementary Figure 6. Gene sets characteristic to subsets of *Onecut3$^+$* neurons. Stacked violin plots for marker genes for ‘top 20’ most divergent genes amongst GABA only, glutamate only, *Th*/GABA and *Trh*/glutamate co-expressing neuron populations[9]. Data were extracted from Ref.[9], using the open-label dataset GSE 132730.

Supplementary Figure 7. Morphological changes after *Onecut3* overexpression in neuro- and glioblastoma cell lines. (a,a_1) Transient overexpression of *Onecut3* transformed Neuro-2a cells to grow elongated neurites, and to upregulate microtubule associated protein, MAP2 (green, arrowheads). Two examples are shown. (b-c_1) In U251 glioblastoma cells, transient overexpression of *Onecut3* induced the immature neuronal marker beta-III tubulin (TUJ1, arrowheads), as well as MAP2 (c_1, solid arrowheads), at the expense glial fibrillary acidic protein (GFAP, red). Note the lack of GFAP in the transfected cells. Scale bars = 20 µm (a-c).

Supplementary Figure 8. *Onecut3* overexpression in neural stem cells. (a) Transient transfection (CMV-OC3) or lentiviral particle-induced transduction of *Onecut3* (LE-OC3) in neurospheres prepared from the hypothalamus (hyp) and cerebrum (ctx), respectively, limited the expansion of neurospheres. (b) The mitotic (proliferation) marker phospho-histone H3 (pHH3) did not co-localize with ONECUT3 (solid arrowheads). Hypothalamic neurospheres are shown. (c) Similarly, ONECUT3$^+$/GFP$^+$ cells did not express SOX2, another proliferation marker, either (solid arrowheads). A

cortical neurosphere is shown. Source data are provided as a Source Data file. *$p < 0.05$, ***$p < 0.001$, Student's t-test, $n > 100$ clusters/condition. Scale bars = 100 µm (a,b), 20 µm (c).

Supplementary Figure 9. Differentially-expressed genes upon *Onecut3* overexpression in Neuro-2a cells. Volcano plot depicting significantly up- or down-regulated genes (red solid circles) upon *Onecut3* overexpression. Data are from bulk RNA-seq (n = 2 biological pools). Note an increased expression of genes involved in neurite outgrowth (*Nav2*, *Robo3*, *Dok5*, *Ephb2*) *vs*. a reduction in genes controlling neural progenitor proliferation (*Ascl2*, *Ovgp1*). Selected genes with adjusted p-value < 0.005 are denoted in red. Minimal expression value = 1; observed $\log_2$FoldChange >2 or <-0.45.

Supplementary Figure 10. Inhibition of Rho-associated kinase (ROCK) amplifies Onecut3 effects on neuritogenesis. (a) Inhibition of ROCK with Y-27632 (5 µM) augmented neurite outgrowth in both Onecut3 transfected (arrowheads) and untransfected cells (open arrowheads). (a_1) Quantitative data from continuous live-cell imaging using an Incucyte SX5 platform. Left: cell proliferation was reduced by either *Onecut3* and *Onecut3* + Y-27632. Right: neurite length was significantly increased by *Onecut-3,* with an additive effect of Y-37632. Data were expressed as average cell body cluster (mm^2/mm^2) and neurite outgrowth length (mm/mm^2) from n = 8 biological replicates per condition. Color-coded shade shows data range per time point (s.e.m.). *$p < 0.05$, **$p < 0.01$, two-way repeated measures ANOVA, n = 4 replicates/condition. Differences between control *vs. Onecut3* are in blue, while control *vs. Onecut3* + Y-27632 appear in red. Source data are provided as a Source Data file. Scale bar = 50 µm (a).

Supplementary Figure 11. Genetic deletion of *ceh-48* in *C. elegans* increases pharynx length. (a) Schema showing the main sensory neurons in the head region of *C. elegans*. (b,c) DiI labeling of a wild type and *unc-53* mutant with measurements of pharynx length (n = 5-8/group). Statistical differences between the groups were tested by using a two-tailed ANOVA (b). Abbreviations; t, terminal bulb; DIC, differential interference contrast. ***$p < 0.001$ *vs*. both wild-type and *unc-53*. Source data are provided as a Source Data file. Scale bar = 20 µm (b).

Supplementary references

1. Tamamaki, N. *et al.* Green fluorescent protein expression and colocalization with calretinin, parvalbumin, and somatostatin in the GAD67-GFP knock-in mouse. *J Comp Neurol* **467**, 60–79 (2003).
2. López-Bendito, G. *et al.* Preferential origin and layer destination of GAD65-GFP cortical interneurons. *Cereb Cortex* **14**, 1122–1133 (2004).
3. Zupančič, M. *et al.* Brain-wide mapping of efferent projections of glutamatergic (Onecut3+) neurons in the lateral mouse hypothalamus. *Acta Physiologica* **n/a**, e13973.
4. Varga, E. *et al.* Thyrotropin-Releasing-Hormone-Synthesizing Neurons of the Hypothalamic Paraventricular Nucleus Are Inhibited by Glycinergic Inputs. *Thyroid* **29**, 1858–1868 (2019).
5. Hippenmeyer, S. *et al.* A developmental switch in the response of DRG neurons to ETS transcription factor signaling. *PLoS Biol* **3**, e159 (2005).
6. Madisen, L. *et al.* A robust and high-throughput Cre reporting and characterization system for the whole mouse brain. *Nat Neurosci* **13**, 133–140 (2010).
7. Espana, A. & Clotman, F. Onecut transcription factors are required for the second phase of development of the A13 dopaminergic nucleus in the mouse. *J Comp Neurol* **520**, 1424–1441 (2012).
8. Mihaly, E., Legradi, G., Fekete, C. & Lechan, R. M. Efferent projections of ProTRH neurons in the ventrolateral periaqueductal gray. *Brain Res* **919**, 185–197 (2001).
9. Romanov, R. A. *et al.* Molecular design of hypothalamus development. *Nature* 1–7 (2020) doi:10.1038/s41586-020-2266-0.

E13.5 in utero electroporation with mCherry, harvested on E15.5 / E18.5

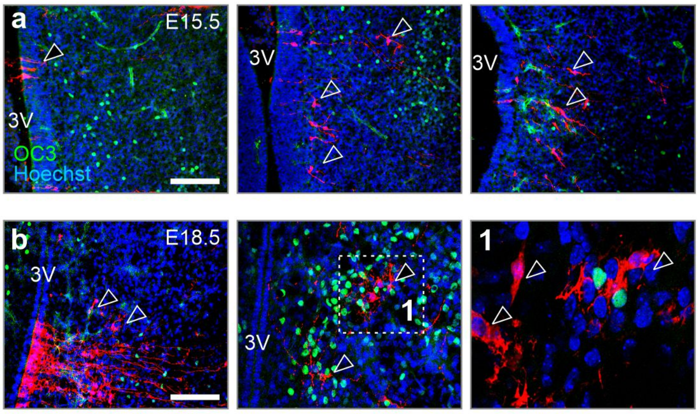

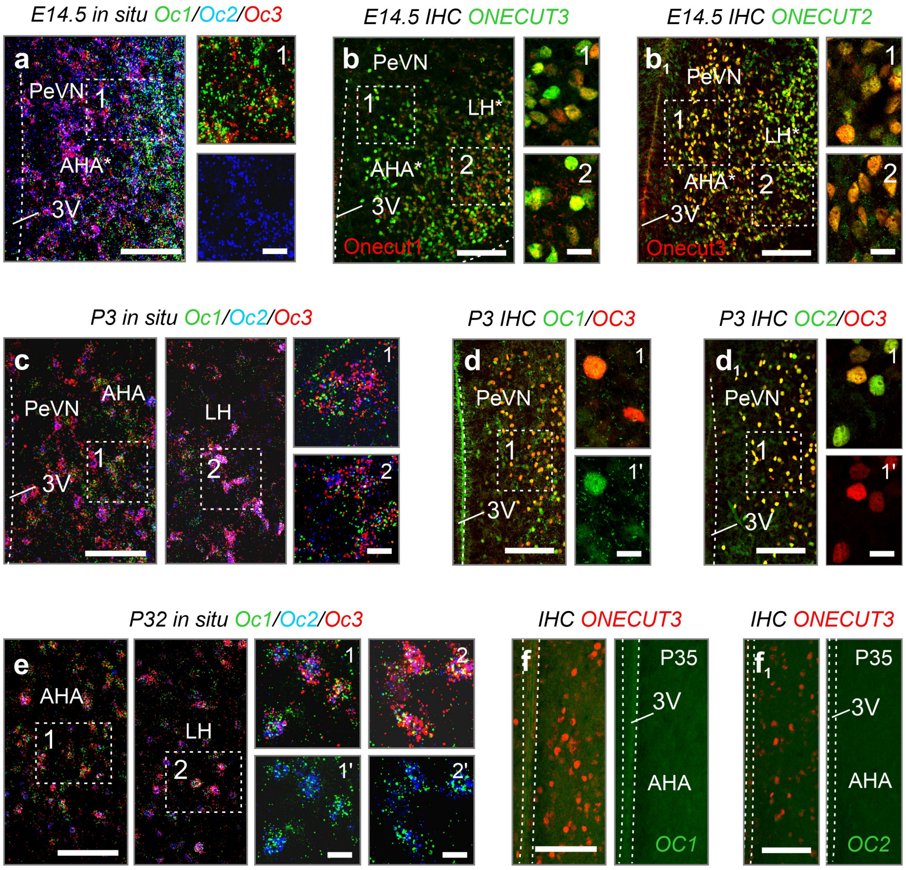

E14.5 in situ Oc1/Oc2/Oc3
E14.5 IHC ONECUT3
E14.5 IHC ONECUT2
a
PeVN
1
AHA*
3V
b
PeVN
LH*
AHA*
2
Onecut1
b1
Onecut3
P3 in situ Oc1/Oc2/Oc3
P3 IHC OC1/OC3
P3 IHC OC2/OC3
c
AHA
LH
d
1'
d1
P32 in situ Oc1/Oc2/Oc3
IHC ONECUT3
IHC ONECUT3
e
2'
f
P35
OC1
f1
OC2

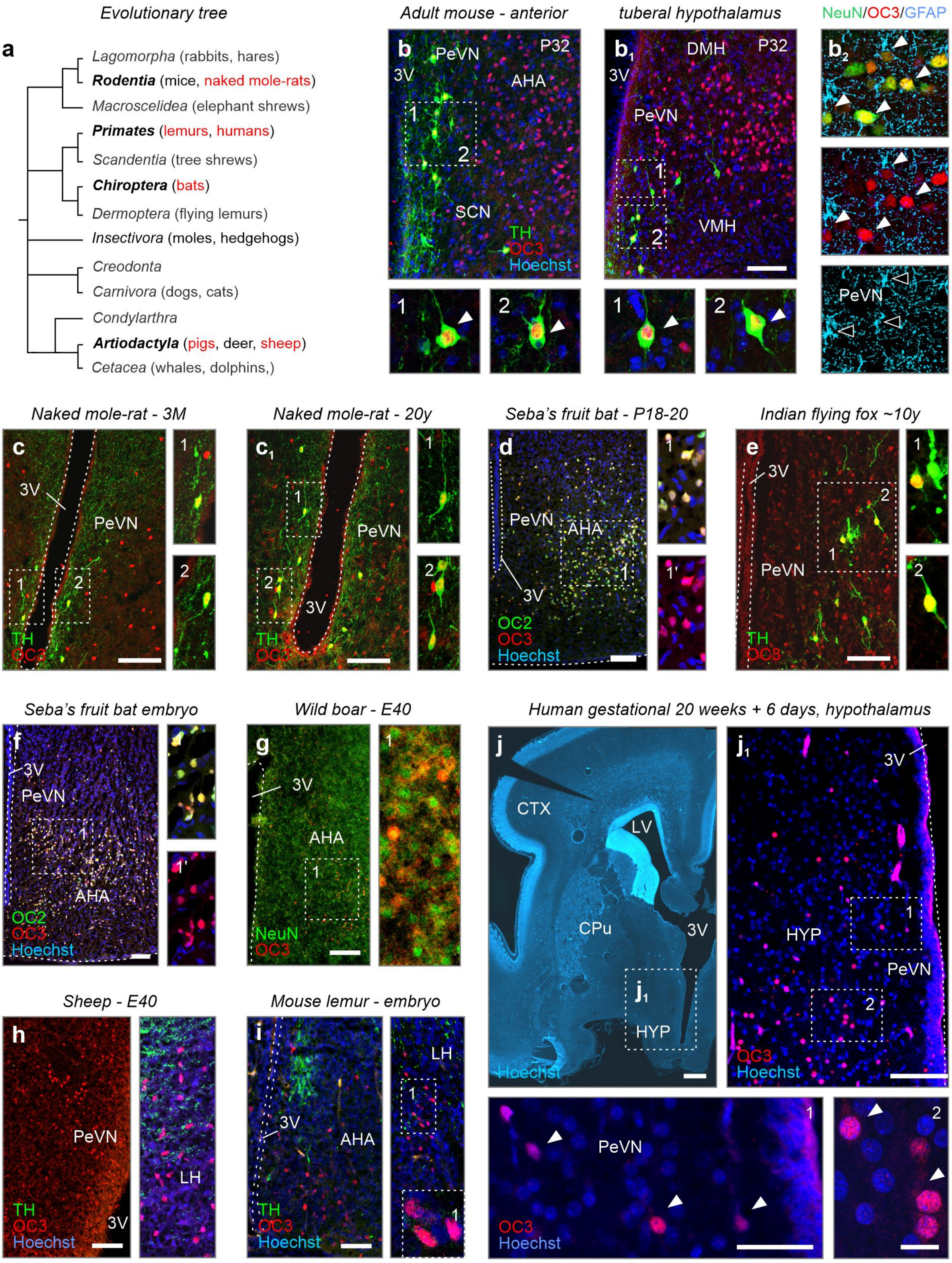

Evolutionary tree
a
Lagomorpha (rabbits, hares)
Rodentia (mice, naked mole-rats)
Macroscelidea (elephant shrews)
Primates (lemurs, humans)
Scandentia (tree shrews)
Chiroptera (bats)
Dermoptera (flying lemurs)
Insectivora (moles, hedgehogs)
Creodonta
Carnivora (dogs, cats)
Condylarthra
Artiodactyla (pigs, deer, sheep)
Cetacea (whales, dolphins,)
Adult mouse - anterior
tuberal hypothalamus
NeuN/OC3/GFAP
b
PeVN
P32
3V
AHA
SCN
TH
OC3
Hoechst
b1
DMH
PeVN
VMH
b2
PeVN
Naked mole-rat - 3M
c
3V
PeVN
TH
OC3
Naked mole-rat - 20y
c1
PeVN
3V
TH
OC3
Seba's fruit bat - P18-20
d
PeVN
AHA
3V
OC2
OC3
Hoechst
Indian flying fox ~10y
e
3V
PeVN
TH
OC3
Seba's fruit bat embryo
f
3V
PeVN
AHA
OC2
OC3
Hoechst
Wild boar - E40
g
3V
AHA
NeuN
OC3
Human gestational 20 weeks + 6 days, hypothalamus
j
CTX
LV
CPu
3V
j1
HYP
Hoechst
j1
3V
HYP
PeVN
OC3
Hoechst
Sheep - E40
h
PeVN
TH
OC3
3V
Hoechst
LH
Mouse lemur - embryo
i
3V
AHA
TH
OC3
Hoechst
LH
PeVN
OC3
Hoechst

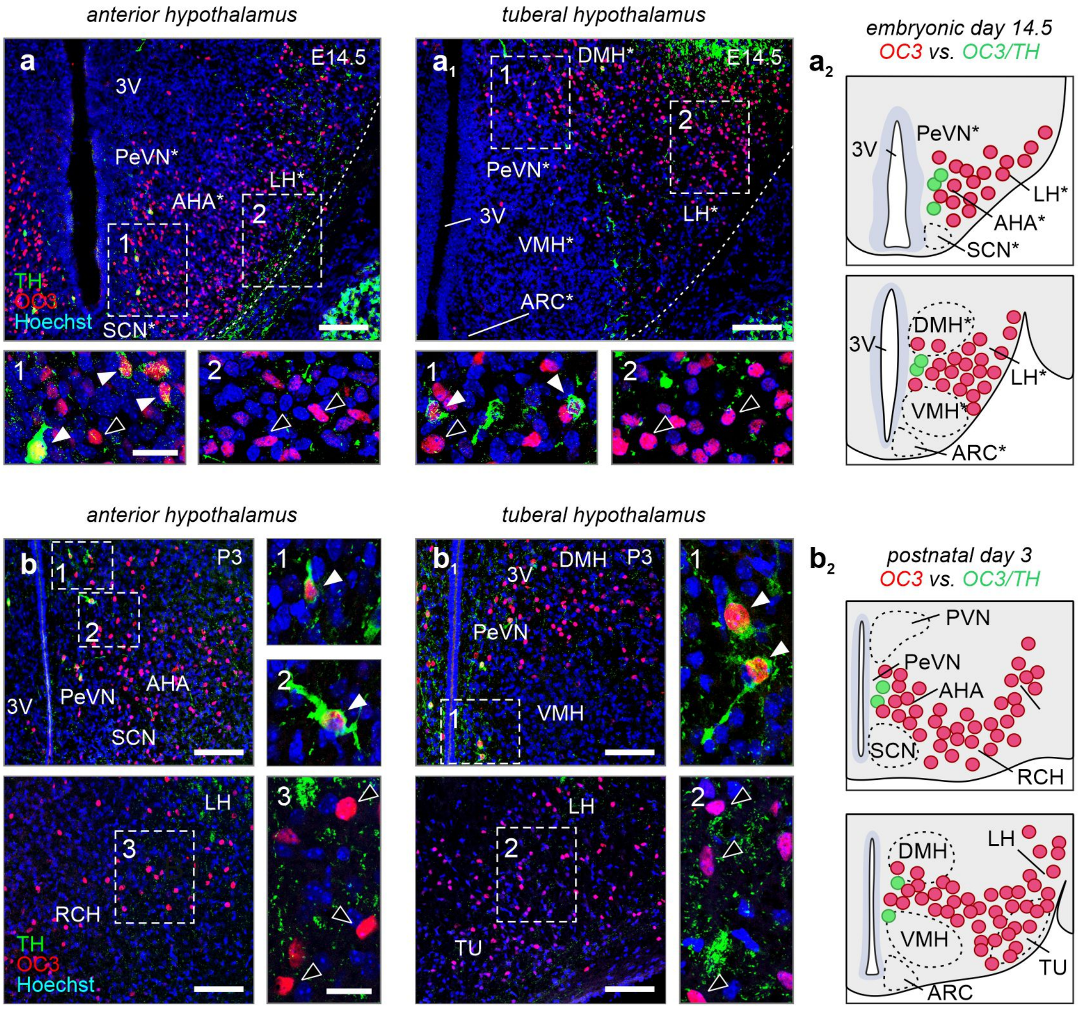
anterior hypothalamus
tuberal hypothalamus
embryonic day 14.5
OC3 vs. OC3/TH
a
E14.5
3V
PeVN*
AHA*
LH*
SCN*
TH
OC3
Hoechst
a1
DMH*
PeVN*
3V
VMH*
ARC*
LH*
a2
PeVN*
LH*
AHA*
SCN*
DMH*
VMH*
ARC*
anterior hypothalamus
tuberal hypothalamus
postnatal day 3
OC3 vs. OC3/TH
b
P3
3V
PeVN
AHA
SCN
LH
RCH
TH
OC3
Hoechst
b1
3V
DMH
PeVN
VMH
LH
TU
b2
PVN
PeVN
AHA
SCN
RCH
DMH
LH
VMH
TU
ARC

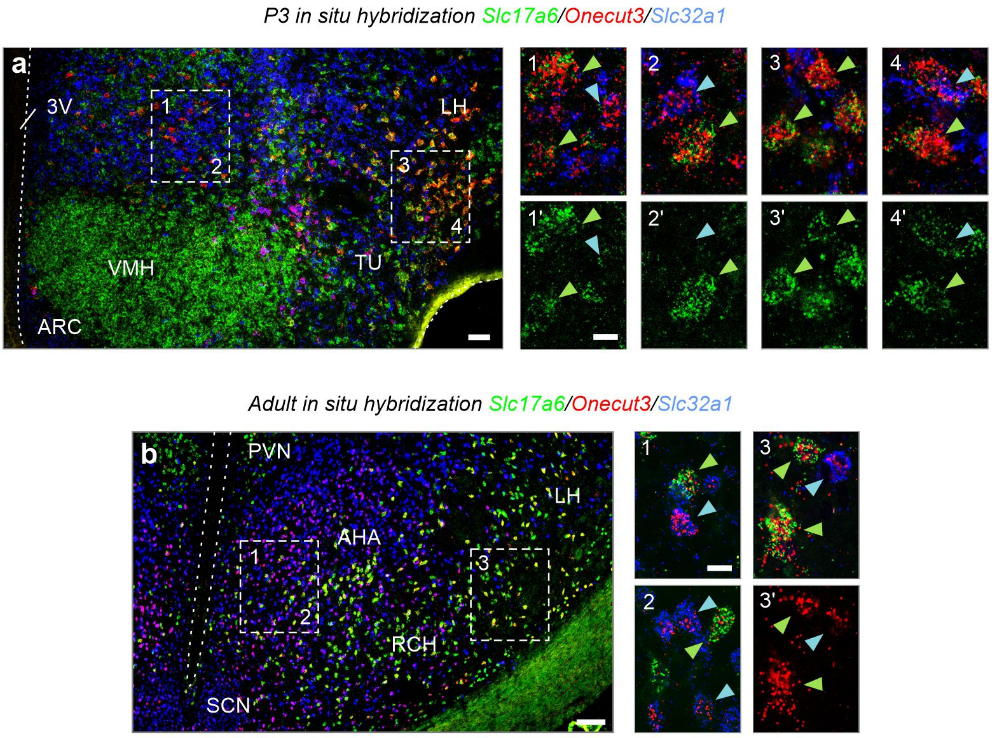
P3 in situ hybridization Slc17a6/Onecut3/Slc32a1
a
3V
1
2
LH
3
4
VMH
TU
ARC
1
2
3
4
1'
2'
3'
4'
Adult in situ hybridization Slc17a6/Onecut3/Slc32a1
b
PVN
LH
AHA
1
2
3
RCH
SCN
1
3
2
3'

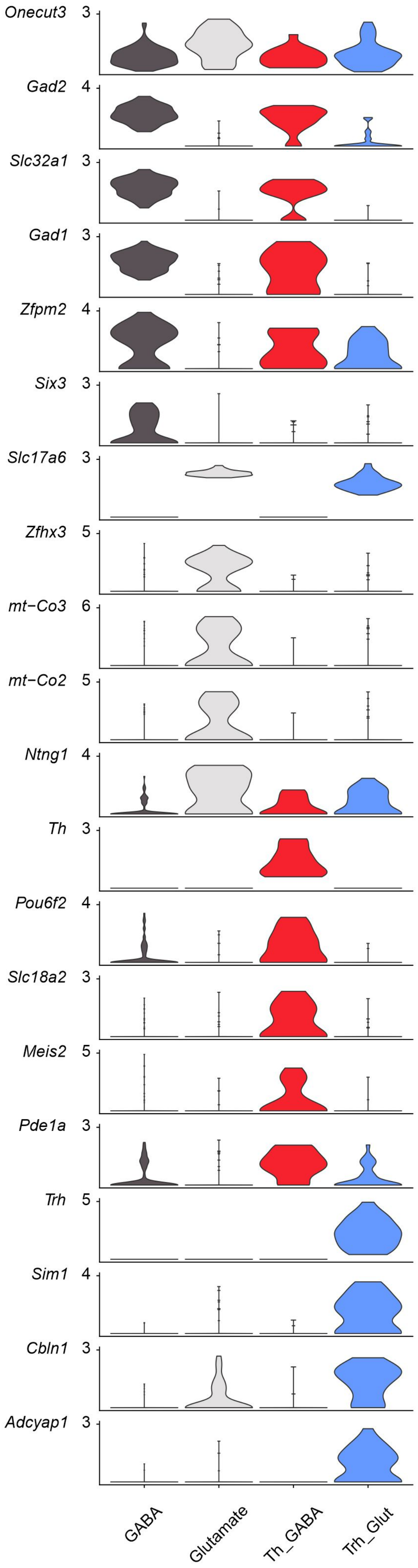
Onecut3
3
Gad2
4
Slc32a1
3
Gad1
3
Zfpm2
4
Six3
3
Slc17a6
3
Zfhx3
5
mt−Co3
6
mt−Co2
5
Ntng1
4
Th
3
Pou6f2
4
Slc18a2
3
Meis2
5
Pde1a
3
Trh
5
Sim1
4
Cbln1
3
Adcyap1
3
GABA
Glutamate
Th_GABA
Trh_Glut

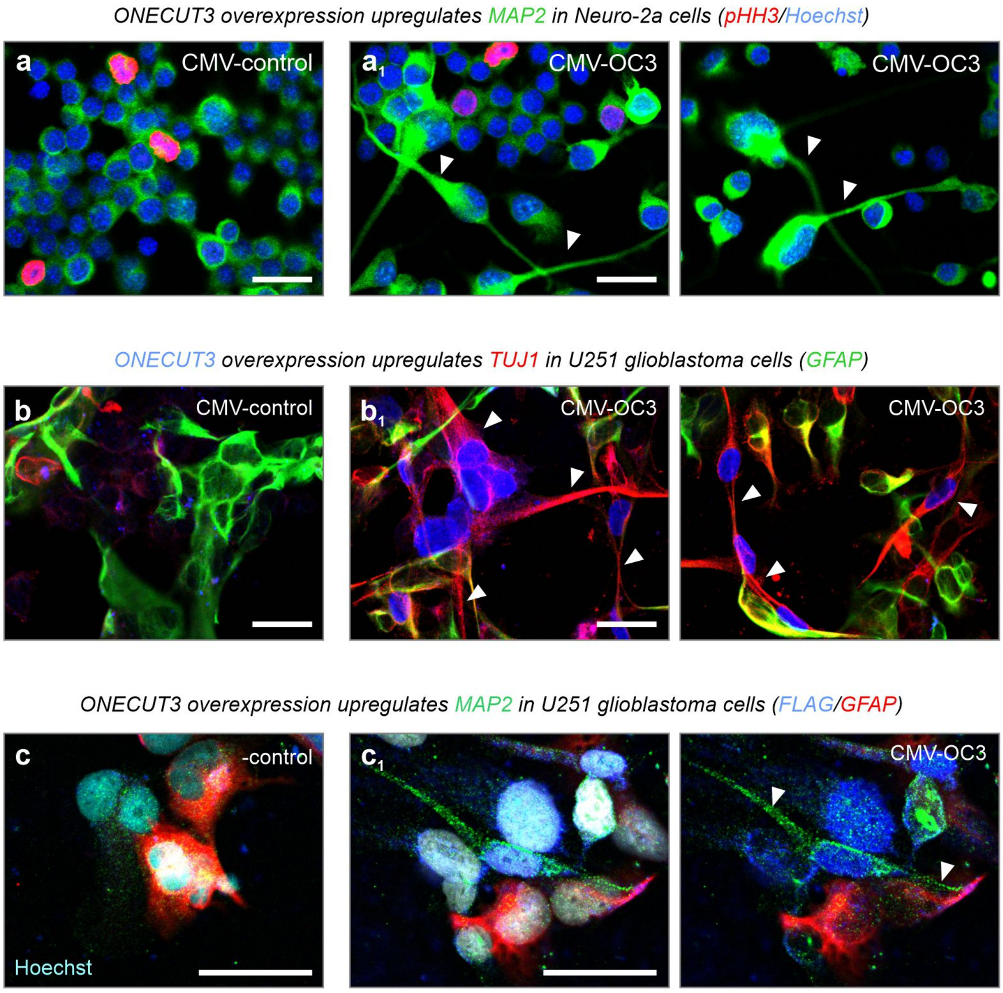
ONECUT3 overexpression upregulates MAP2 in Neuro-2a cells (pHH3/Hoechst)
a
CMV-control
a1
CMV-OC3
CMV-OC3
ONECUT3 overexpression upregulates TUJ1 in U251 glioblastoma cells (GFAP)
b
CMV-control
b1
CMV-OC3
CMV-OC3
ONECUT3 overexpression upregulates MAP2 in U251 glioblastoma cells (FLAG/GFAP)
c
-control
Hoechst
c1
CMV-OC3

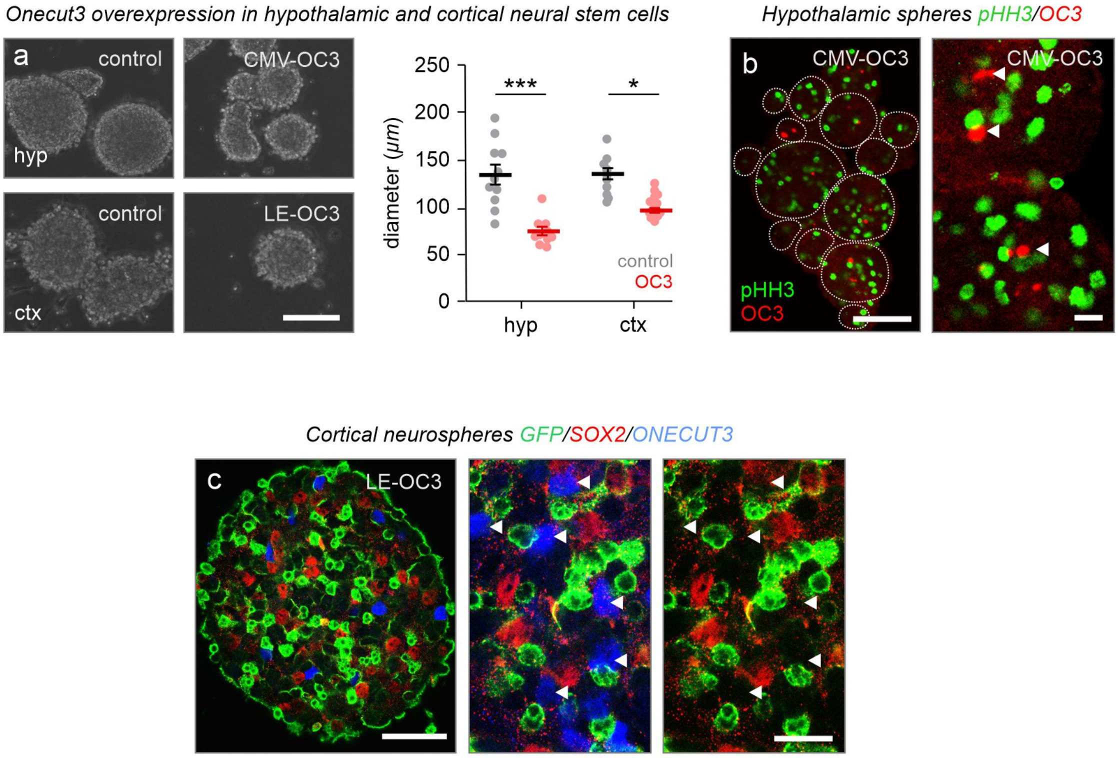
Onecut3 overexpression in hypothalamic and cortical neural stem cells
Hypothalamic spheres pHH3/OC3
a
control
CMV-OC3
hyp
control
LE-OC3
ctx
250
200
150
100
50
0
diameter (µm)
***
*
control
OC3
hyp
ctx
b
CMV-OC3
CMV-OC3
pHH3
OC3
Cortical neurospheres GFP/SOX2/ONECUT3
c
LE-OC3

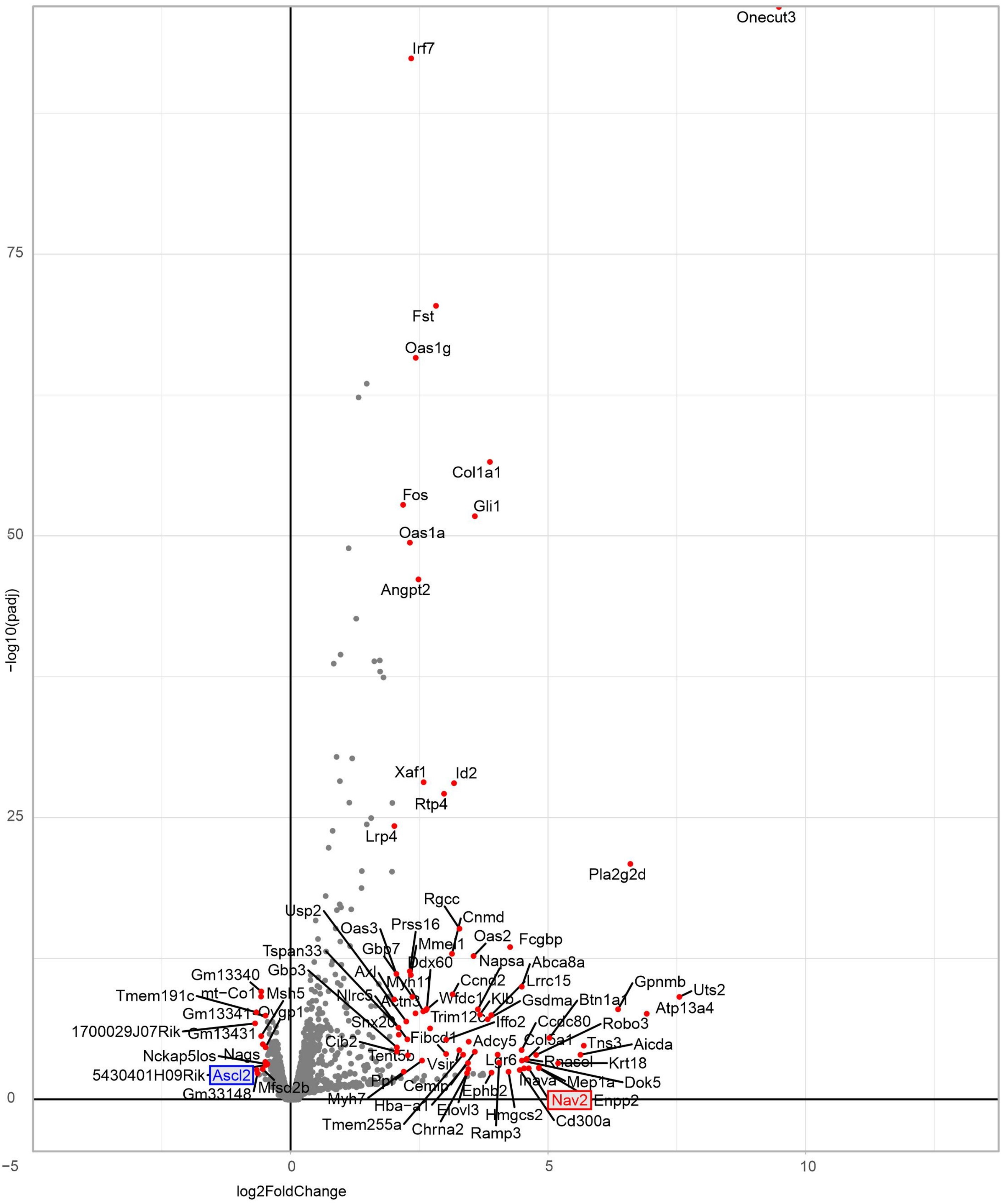
Onecut3
Irf7
Fst
Oas1g
Col1a1
Fos
Gli1
Oas1a
Angpt2
Xaf1
Id2
Rtp4
Lrp4
Pla2g2d
Rgcc
Cnmd
Usp2
Oas3
Prss16
Oas2
Fcgbp
Gbp7
Mme
Tspan33
Ddx60
Napsa
Abca8a
Gbp3
Axl
Myh11
Ccnd2
Lrrc15
Gpnmb
Uts2
Gm13340
Tmem191c
mt−Co1
Msh5
Nlrc5
Actn3
Wfdc1
Klb
Gsdma
Btn1a1
Atp13a4
Gm13341
Ovgp1
Trim12c
Iffo2
Snx20
Ccdc80
Robo3
1700029J07Rik
Gm13431
Fibcd1
Adcy5
Col5a1
Tns3
Aicda
Cib2
Nckap5los
Nags
Tent5b
Vsir
Lgr6
Rnasel
Krt18
5430401H09Rik
Ascl2
Ppl
Inava
Mep1a
Dok5
Gm33148
Mfsd2b
Cemip
Ephb2
Nav2
Enpp2
Myh7
Hba−a1
Elovl3
Hmgcs2
Cd300a
Tmem255a
Chrna2
Ramp3
75
50
25
0
−5
0
5
10
−log10(padj)
log2FoldChange

Y-27632 effects on Neuro-2a cells overexpressing ONECUT3

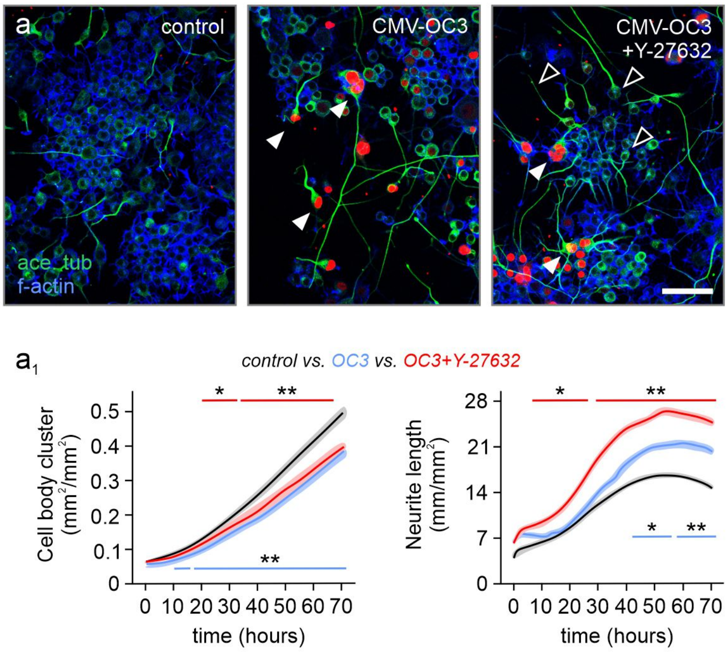

C. elegans DiI neuronal labeling and measurements

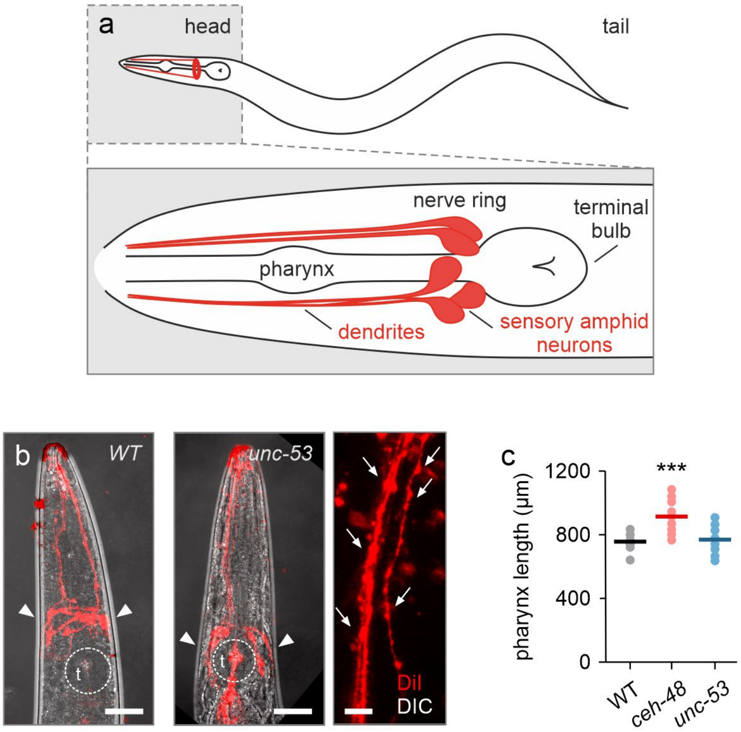

Supplement: Supplementary file 1 — Supplementary Information [file 41467_2024_52762_MOESM1_ESM.pdf]
