# Supplementary material for: Concerted transcriptional regulation of the morphogenesis of hypothalamic neurons by ONECUT3
Source: Nat Commun. 2024 Oct 5;15:8631. doi: 10.1038/s41467-024-52762-z (PMC11452682; doi:10.1038/s41467-024-52762-z)
Supplement: Supplementary file 3 — Description of Additional Supplementary Files [file 41467_2024_52762_MOESM3_ESM.pdf]

Description of Additional Supplementary Information

Supplementary Movies 1-4: Live-cell IncuCyte imaging of Neuro-2a cells showing increased neurite outgrowth and migration when overexpressing ONECUT3. Treatment with the TRIO inhibitor ITX3 significantly reduces this response.

Supplementary Data 1: List of gene expression changes after ONECUT3 overexpression in Neuro-2a cells.
