# Supplementary material for: Concerted transcriptional regulation of the morphogenesis of hypothalamic neurons by ONECUT3
Source: Nat Commun. 2024 Oct 5;15:8631. doi: 10.1038/s41467-024-52762-z (PMC11452682; doi:10.1038/s41467-024-52762-z)
Supplement: Supplementary file 9 — Reporting Summary [file 41467_2024_52762_MOESM9_ESM.pdf]

nature portfolio

Corresponding author(s): Tibor HARKANY

Last updated by author(s): May 3, 2024

Reporting Summary

Nature Portfolio wishes to improve the reproducibility of the work that we publish. This form provides structure for consistency and transparency in reporting. For further information on Nature Portfolio policies, see our Editorial Policies and the Editorial Policy Checklist.

Statistics

For all statistical analyses, confirm that the following items are present in the figure legend, table legend, main text, or Methods section.

n/a	Confirmed	
☐	☒	The exact sample size (*n*) for each experimental group/condition, given as a discrete number and unit of measurement
☐	☒	A statement on whether measurements were taken from distinct samples or whether the same sample was measured repeatedly
☐	☒	The statistical test(s) used AND whether they are one- or two-sided<br>*Only common tests should be described solely by name; describe more complex techniques in the Methods section.*
☐	☒	A description of all covariates tested
☒	☐	A description of any assumptions or corrections, such as tests of normality and adjustment for multiple comparisons
☐	☒	A full description of the statistical parameters including central tendency (e.g. means) or other basic estimates (e.g. regression coefficient) AND variation (e.g. standard deviation) or associated estimates of uncertainty (e.g. confidence intervals)
☐	☒	For null hypothesis testing, the test statistic (e.g. *F*, *t*, *r*) with confidence intervals, effect sizes, degrees of freedom and *P* value noted<br>*Give P values as exact values whenever suitable.*
☒	☐	For Bayesian analysis, information on the choice of priors and Markov chain Monte Carlo settings
☒	☐	For hierarchical and complex designs, identification of the appropriate level for tests and full reporting of outcomes
☒	☐	Estimates of effect sizes (e.g. Cohen's *d*, Pearson's *r*), indicating how they were calculated

Our web collection on statistics for biologists contains articles on many of the points above.

Software and code

Policy information about availability of computer code

Data collection	1) Confocal image acquisition: (a) LSM 710 confocal laser-scanning microscope (Zeiss). (b) LSM 880 confocal laser-scanning microscope with Airyscan Detector (Zeiss). (c) LSM 900 confocal laser-scanning microscope with Airyscan 2 Detector (Zeiss). 2) Image analysis: (a) Imaris x64 9.0.2 (Bitplane). (b) Fiji 1.52e (GNU General Public Licence, https://imagej.net/Fiji). 3) RT-qPCR data: (a) CFX-connect (BioRad). 4) snRNA-seq: Libraries prepared with the Chromium Next GEM Single Cell 3' Reagent Kit v3.1 (10x Genomics #PN1000128) were sequenced using an Illumina HiSeq 3000/HiSeq 4000 System with corresponding Illumina commercial software (HCS v3.4.0, RRID:SCR_016386). Sequences were obtained using an Illumina HiSeq 3000/HiSeq 4000 System with corresponding Illumina commercial software (HCS v3.4.0, RRID:SCR_016386); scRNA-seq data used in this study were previously deposited in raw and processed forms to the NCBI Gene Expression Omnibus, with accession number (GSE132730). For imaging, ZEN2010 (RRID:SCR_013672) was used as control software for a Zeiss LSM880 laser-scanning microscope.
Data analysis	Cell Ranger (v7.1.0, RRID:SCR_017344), ZEN (Black, 2022, RRID:SCR_013672), ImageJ (v1.49, RRID:SCR_003070), GraphPad Prism (v8, RRID:SCR_002798) were used, all in commercially-available configurations without the introduction of custom-made codes in our laboratory. We used existing R packages (RRID:SCR_001905) and Python modules (RRID:SCR_008394) as outlined in the on-line resource (https://harkany-lab.github.io/Zupancic_2023:). All required software and configurations are additionally available in Docker image at https://hub.docker.com/layers/etretiakov/workbench-session-complete/jammy-2023.04.08-custom-11.6/images/sha256-431a998497b7169b970a90398412bb91dbc99b37403d27f8ca98b3ace66db0ba?context=explore that were used for rendering of analysis notebooks; images can be obtained using bash command: `docker pull etretiakov/workbench-session-complete:jammy-2023.04.08-custom-11.6.

For manuscripts utilizing custom algorithms or software that are central to the research but not yet described in published literature, software must be made available to editors and reviewers. We strongly encourage code deposition in a community repository (e.g. GitHub). See the Nature Portfolio guidelines for submitting code & software for further information.

Data

Policy information about availability of data

All manuscripts must include a data availability statement. This statement should provide the following information, where applicable:

- Accession codes, unique identifiers, or web links for publicly available datasets
- A description of any restrictions on data availability
- For clinical datasets or third party data, please ensure that the statement adheres to our policy

Data for single-cell and bulk RNA-seq were archived at https://harkany-lab.github.io/Zupancic_2023; https://github.com/Harkany-Lab/Zupancic_2023 and deposited to Figshare.com with DOI: 10.6084/m9.figshare.22680433. All other data are available in the source data file.

Research involving human participants, their data, or biological material

Policy information about studies with human participants or human data. See also policy information about sex, gender (identity/presentation), and sexual orientation and race, ethnicity and racism.

Reporting on sex and gender	Tissues were obtained from mixed backgrounds
Reporting on race, ethnicity, or other socially relevant groupings	Tissues were obtained from mixed backgrounds
Population characteristics	Tissues were obtained from mixed backgrounds
Recruitment	Tissues were provided randomly from healthy aborted fetuses
Ethics oversight	Ethical approval number: 1316/2012, Medical University of Vienna

Note that full information on the approval of the study protocol must also be provided in the manuscript.

Field-specific reporting

Please select the one below that is the best fit for your research. If you are not sure, read the appropriate sections before making your selection.

☒ Life sciences ☐ Behavioural & social sciences ☐ Ecological, evolutionary & environmental sciences

For a reference copy of the document with all sections, see nature.com/documents/nr-reporting-summary-flat.pdf

Life sciences study design

All studies must disclose on these points even when the disclosure is negative.

Sample size	Sample size was chosen based on the available embryos coming out of our pregnancies, or when experiments were confirmed for desired genotypes. All sample sizes are mentioned in the legends.
Data exclusions	No data were excluded from the analysis.
Replication	The experiments reported here were minimally performed in duplicates (biological repeats in two (or more) independent experimental settings). All attempts of replication were successful.
Randomization	Animals were selected random to homogenize individual variances
Blinding	Experimenters were not blinded to the experimental conditions because control and treatment groups were simultaneously tested throughout (minimally n > 3/group at any given time), and processed automatically to prevent experimental bias. Thus, blinding was not considered as a factor of objectivity.

Reporting for specific materials, systems and methods

We require information from authors about some types of materials, experimental systems and methods used in many studies. Here, indicate whether each material, system or method listed is relevant to your study. If you are not sure if a list item applies to your research, read the appropriate section before selecting a response.

Materials & experimental systems

n/a	Involved in the study
	☒ Antibodies
	☒ Eukaryotic cell lines
☒	☐ Palaeontology and archaeology
	☒ Animals and other organisms
☒	☐ Clinical data
☒	☐ Dual use research of concern
☒	☐ Plants

Methods

n/a	Involved in the study
☒	☐ ChIP-seq
☒	☐ Flow cytometry
☒	☐ MRI-based neuroimaging

Antibodies

Antibodies used

Onecut3 (F. Clotman; 1:5000) Onecut1 (R&D systems, #AF6277, CCOW0120011; 1:50); Onecut2 (R&D systems, #AF6294, CCOW0118041; 1:50); Sox2 (Abcam, # ab97959, GR3427835-1; 1:500); Gap43 (Millipore, #AB5220, 3510861; 1:500); CPCA-mCherry (EnCor, #CPCA-mCherry, 7670-4; 1:1000); GFP-FITC (Abcam, #ab6662, GR3286332-5; 1:1000), NeuN (Millipore, #MAB377, 3574318; 1:1000); TH (Millipore, #AB152, 3753287; 1:500); Pro-TRH (C. Fekete; 1:200); Acetylated tubulin (Sigma, #T7451; 1:1000); Nav2 (Novus Bio, #NBP1-84615, R01718; 1:1000); Doublecortin (Millipore, #AB2253; 1:1000); GFAP (Synaptic Systems, #173002, 3-38; 1:1000); Map2 (Synaptic Systems, #1880040; 1:1000); Tuj1 (Promega, # G7121, 0000372576; 1:2000); Phosphohistone H3 (Cell Signalling, #9701, 7; 1:500).

Validation

Antibodies have been either validated by the provider, or extensively tested and approved by the scientific community. For this, references are available on the suppliers web site. For full information on antibodies used, please see table 4 of our supporting information.

Eukaryotic cell lines

Policy information about cell lines and Sex and Gender in Research

Cell line source(s)

ATCC

Authentication

Cells lines were authenticated by the provider

Mycoplasma contamination

Negative

Commonly misidentified lines
(See ICLAC register)

Name any commonly misidentified cell lines used in the study and provide a rationale for their use.

Animals and other research organisms

Policy information about studies involving animals; ARRIVE guidelines recommended for reporting animal research, and Sex and Gender in Research

Laboratory animals

Mouse, c57bl6/J, GAD67-GFP, GAD65-GFP, Onecut3-iCRE, Onecut3-mCherry, TRH-tdTomato, Tau-mGFP, Ai14-tdTomato;
C. elegans, N2, mt152, tm237, of all ages ranging from E8.5 until adulthood. Embryonic, neonates and adult, naked mole rats, Seba's fruit bats and Indian flying foxes were obtained from Schönbrunn Zoo (Vienna, Austria) and their use approved by the Austrian Ministry of Science and Research. Fetal wild boar and sheep tissue collection was approved by the German Centre for the Protection of Laboratory Animals, and processed by Simone Fietz and Wolfgang Härtig (University of Leipzig).

Wild animals

No wild animals were used.

Reporting on sex

Both males and females were used in this study

Field-collected samples

No field-collection took place.

Ethics oversight

Experimental procedures on mice conformed to the 2010/63/EU directive and were approved by the Austrian Ministry of Education, Science and Research (66.009/0145-WF/II/3b/2014 and 66.009/0277-WF/V3b/2017). All procedures were planned to reduce suffering, as well as animal numbers.

Note that full information on the approval of the study protocol must also be provided in the manuscript.

Plants

Seed stocks	N/A
Novel plant genotypes	N/A
Authentication	N/A
